# High-risk human papillomavirus genotyping in women with atypical squamous cells of undetermined significance

**DOI:** 10.1038/s41598-023-39206-2

**Published:** 2023-07-26

**Authors:** Pornporm Ittiamornlert, Nida Jareemit, Rattiya Phianpiset, Sompop Kuljarusnont, Suchanan Hanamornroongruang, Navin Horthongkham, Pornnida Khajorndumrongcherdkul, Irene Ruengkhachorn

**Affiliations:** 1 Division of Gynecologic Oncology, Department of Obstetrics and Gynecology, Faculty of Medicine Siriraj Hospital, Mahidol University, 2 Wanglang Road, Bangkoknoi, Bangkok, 10700 Thailand; 2 Division of Gynecologic Oncology, Department of Obstetrics and Gynecology, Chulabhorn Royal Academy, Bangkok, Thailand; 3 Division of Gynecologic Oncology, Department of Obstetrics and Gynecology, Faculty of Medicine, Chiang Mai University, Chiang Mai, Thailand; 4 Department of Pathology, Faculty of Medicine Siriraj Hospital, Mahidol University, Bangkok, Thailand; 5 Department of Microbiology, Faculty of Medicine Siriraj Hospital, Mahidol University, Bangkok, Thailand; 6 Division of Obstetrics and Gynaecological Nursing, Department of Nursing Siriraj Hospital, Mahidol University, Bangkok, Thailand

**Keywords:** Cancer, Biomarkers, Diseases, Oncology, Risk factors

## Abstract

We conducted a prospective study to evaluate the prevalence of high-risk human papillomavirus (hr-HPV) positivity in women with atypical squamous cells of undetermined significance (ASC-US). Additionally, we assessed the association of hr-HPV positivity with the pathology of high-grade squamous intraepithelial lesions or worse (HSIL^+^) and the risk of subsequent detection of squamous intraepithelial lesions. A total of 376 women were included, with 242 (64.4%) exhibiting hr-HPV positivity. The predominant HPV genotypes were 16, 52 and 58. Factors associated with the immediate detection of HSIL^+^ pathology included a colposcopic impression of high-grade lesions, hr-HPV positivity, HPV 16 positivity, HPV 18 positivity, HPV 58 positivity, age less than 40 years, and biopsy of two or more pieces. However, only the first three factors were statistically significant in multivariate analysis. Among the 291 women who continued surveillance for 6 months or more, the median follow-up period was 41.8 months (interquartile range [IQR] 26.5–54.0). The prevalence of subsequent HSIL in women with hr-HPV positivity versus negativity was 3.6% versus 0.98%, respectively. The median time to the subsequent detection of SIL was 28.7 months (IQR 14.9–41.7). In conclusion, women with ASC-US in our study had a high proportion of hr-HPV positivity. Type-specific HPV testing could play a pivotal role in the development of specific management protocols for women with ASC-US.

*Clinical trial registration*: https://thaiclinicaltrials.org, TCTR20161017002.

## Introduction

Cervical cancer ranks as the fourth most common cancer and the fourth leading cause of cancer-related death in women globally, with 56.8% of cervical cancer cases resulting in mortality^1^. The screening tests available include (i) high-risk human papillomavirus (hr-HPV) DNA/mRNA tests, (ii) cytology, (iii) co-testing, and (iv) visual inspection with acetic acid (VIA)^2,3^. In Thailand, cytology screening remains principal approach, yielding abnormal results in 1.5–4% of cases. The most frequently identified cytological abnormality is atypical squamous cells of undetermined significance (ASC-US)^4,5^.

According to 2019 American Society for Colposcopy and Cervical Pathology (ASCCP) consensus on risk-based management, ASC-US with negative hr-HPV should be followed by a co-testing evaluation after three years. However, ASC-US cases with positive hr-HPV without genotype-specific strategy, necessitate as immediate colposcopy^6^. In a prior report involving 1756 Thai women with ASC-US, 221 (12.6%) demonstrated immediate pathology of high-grade squamous intraepithelial lesion (HSIL) or adenocarcinoma in situ (AIS) or invasive cancer identified in 41 women (2.3%)^7^. Given such a high incidence of immediate HSIL or worse (HSIL^+^), the direct costs of colposcopy are borne by government public health security schemes, rending immediate colposcopy the primary triage method for Thai women with ASC-US.

Different types of hr-HPV exhibit varied oncogenicity. The two types accepted worldwide as the most oncogenic are HPV 16 and 18^8–10^. Geographic variation exists concerning the most common hr-HPV genotypes associated with HSIL^+^ pathology in women with ASC-US. Black women with ASC-US have significantly higher hr-HPV positivity rates and lower HPV 16 positivity than white women^11^. Two studies reported that the most common HPV types detected in women with ASC-US were types 16, 58, and 52, in descending order of prevalence^12,13^. Conversely, another study listed HPV types 16, 18, and 31 as the most common, in that order^14^. An additional study indicated that HPV types 16, 33, 58, and 51 were most associated with detected HSIL^+^^15^. The presence of specific hr-HPV types may significantly impact the triage of women with ASC-US during diagnostic colposcopy.

The aims of this study were to compare the proportions of HSIL^+^ pathology among women with ASC-US cytology for each hr-HPV genotype, to identify factors that are associated with immediate HSIL^+^ pathology, and to investigate the prevalence of subsequent squamous intraepithelial lesion (SIL) pathology during follow-up and explore its associated factors.

## Subjects and methods

Before this research begun, the Siriraj Institutional Review Board authorized the protocol (approval number Si 615/2016). The work was also registered at the Thai Clinical Trials Registry (TCTR20161017002). Informed consent was obtained from all participants. Enrollment of study subjects occurred between March 2016 and March 2018. The authors confirm that all methods were performed in accordance with relevant guidelines and regulations. The inclusion criteria for this study included non-pregnant women aged 21 years or older presenting with ASC-US cytology. Participants were required to have no history of abnormal cervical cytology or treatment for cervical neoplasia, and to provide written informed consent before attending the colposcopy clinic.

Colposcopy was performed within six weeks by gynecologic oncologists. At the beginning step of the colposcopy, a cervical smear was collected for hr-HPV DNA testing using a Wallach broom and preserved in PreservCyt solution (Hologic, Marlborough, MA, USA). For the detection of L1 DNA, we utilized the Anyplex II hr-HPV Detection Kit (Seegene Inc, Seoul, Korea). This kit has been validated by the European Commission and is capable of detecting 14 hr-HPV genotypes: HPV 16, 18, 31, 33, 35, 39, 45, 51, 52, 56, 58, 59, 66, and 68. Colposcopic impressions were classified into three categories: *normal*–lacking acetowhite epithelium or other abnormalities, *low-grade*—thin acetowhite lesion or rapidly fading acetowhitening or fine type of abnormal vessels, and *high-grade*—rapid appearance and dense acetowhitening, coarse type of abnormal vessels or sharp border. Up to four colposcopy-directed biopsies were obtained from all visible lesions, including those of the cervix, vagina, and vulva. If the squamocolumnar junction (SCJ) appeared normal, a random biopsy was performed at 12 o’clock. Endocervical curettage (ECC) was performed when the SCJ could not be fully visualized. Pathologic diagnoses were made by a gynecologic pathologist (SH), according to the 2012 classification of cervical pathology by the International Federation for Cervical Pathology and Colposcopy (IFCPC). Women with HSIL^+^ pathology underwent treatment following standard protocols. On the other hand, women diagnosed with LSIL pathology or less were scheduled for repeating cervical cytology every six months for two years or hr-HPV based-testing at one and two years. Subsequent routine screening was scheduled for women who showed all negative tests. During the follow-up period, the threshold for colposcopy was any abnormal cytology or positive hr-HPV.

Demographic data, results of the hr-HPV DNA testing, colposcopy findings, and pathology reports were collected. The proportion of HSIL^+^ pathology, including HSIL, AIS, or cancer was calculated. Follow-up outcomes were collected until September 30, 2022, and participants who completed follow-up tests for six months or longer were analyzed for subsequent detection of SIL.

The sample size calculation was based on the prevalence of hr-HPV detection in women with ASC-US, which stood as 56%^16^. With a delta error of 0.05, a total of 376 women were required for this study.

PASW Statistics, version 18 (SPSS Inc. Chicago, IL, USA), was employed for all statistical analyses. The data are presented as number and percentage or median and interquartile range (IQR). The Chi-square test or Fisher’s exact test was used to compare categorical variables. Stepwise logistic regression analysis was conducted to identify variables independently associated with HSIL^+^ pathology. The disease-free interval analysis was performed using the Kaplan–Meier method and Cox proportional hazards model. The results of associated factors are reported as odds ratios (OR) with corresponding 95% confidence interval (CIs). A *P* value of 0.05 or less was considered to be statistically significant.

## Results

In total, we enrolled 376 women. Baseline demographic data, results of hr-HPV DNA testing, colposcopy impressions, and pathological report are presented in Table 1. The median age was 40.5 years (IQR 32.0–49.0). None of the study participants had received an HPV vaccination. The proportion of positive hr-HPV was 64.4%. The common HPV genotypes were HPV 16, 52, 58, and 51, respectively. Single HPV genotype infection was found in 168 women, the most common genotypes being HPV 16, 52, 51, and 58, respectively. The two most common HPV genotypes coexisting in multiple infections were HPV 16 and 58. Only one woman diagnosed with squamous cell carcinoma (SCCA), was detected with a single infection by HPV 18.

**Table 1:** Clinical and pathological reports of 376 women with cervical cytology screening results of atypical squamous cells of undetermined significance.

Characteristics	Number (%)
Age (years), median [IQR]	40.5 [32.0–49.0]
Status of hr-HPV	
Negative hr-HPV	134 (35.6)
Positive hr-HPV	242 (64.4)
Single genotypes	168
Multiple genotypes	74
HPV types, n = 242	
HPV 16	64
HPV 52	51
HPV 58	44
HPV 51	28
HPV 18	21
HPV 39	21
HPV 68	21
HPV 66	19
HPV 31	15
HPV 56	10
HPV 35	8
HPV 33	7
HPV 45	4
HPV 59	4
Colposcopic impression	
Normal	82 (21.8)
Low-grade lesion	243 (64.6)
High-grade lesion	49 (13.0)
Inadequate or inconclusive	2 (0.5)
Pathology	
Normal, metaplasia, atrophy	117 (31.1)
Cervicitis	59 (15.7)
LSIL	145 (38.6)
HSIL	54 (14.4)
Invasive cancer	1 (0.3)
Follow-up for ≥ 6 months, n = 291	
Subsequence SIL detection	22 (7.6)
LSIL	14
HSIL	8

*hr-HPV* High-risk human papillomavirus; *HSIL* High-grade squamous intraepithelial lesion; *IQR* Interquartile range; *LSIL* Low-grade squamous intraepithelial lesion.

Immediate HSIL^+^ pathology was found in 55 women (14.7%). No woman was diagnosed with AIS. In the case of the one woman who was diagnosed with SCCA, her conization specimen pathology revealed a depth of invasion of 1 mm without lymphovascular space invasion (LVSI). As a result, she underwent a hysterectomy, and the subsequent pathology report indicated no residual cancer. Among the 54 women diagnosed with HSIL pathology, treatment was successfully completed through cryotherapy, laser ablation, or loop electrosurgical excision procedure (LEEP). One of 54 women with HSIL pathology underwent subsequent hysterectomy and upper vaginectomy due to not-free conization margins and confirmed vaginal HSIL. Factors associated with immediate HSIL^+^ pathology by univariate analysis are presented in Table 2. Multivariate analysis revealed that independent factors significantly associated with immediate HSIL^+^ pathology were a colposcopy impression of high-grade lesions (OR 35.26, 95% CI 4.44–279.83, *P* = 0.001), positive hr-HPV (OR 3.91, 95% CI 1.43–10.64, *P* = 0.008), and positive HPV 16 (OR 2.19, 95% CI 1.08–4.44, *P* = 0.029).

**Table 2:** Associated factors for the pathology of 55 study women who diagnosed with a high-grade squamous intraepithelial lesion or invasive cancer (HSIL^+^).

Variables	HSIL^+^, N (%)	Odds ratio [95% confidence interval]	*P*
Age (years)			
< 40, n = 176	33 (18.8)	Reference	
≥ 40, n = 200	22 (11.0)	0.54 (0.29–0.96)	0.034
HPV status			
hr-HPV status			
Negative hr-HPV, n = 134	5 (3.7)	Reference	
Positive hr-HPV, n = 242	50 (20.7)	6.72 (2.61–17.30)	< 0.001
HPV 16			
Negative, n = 312	35 (11.2)	Reference	
Positive, n = 64	20 (31.3)	3.59 (1.91–6.79)	< 0.001
HPV 18			
Negative, n = 355	49 (13.8)	Reference	
Positive, n = 21	6 (28.6)	2.49 (0.93–6.75)	0.102
HPV 31			
Negative, n = 361	52 (14.4)	Reference	
Positive, n = 15	3 (20.0)	1.49 (0.41–5.45)	0.469
HPV 33			
Negative, n = 369	54 (14.6)	Reference	
Positive, n = 7	1 (14.3)	0.97 (0.12–8.24)	1.000
HPV 35			
Negative, n = 368	55 (15.0)	Reference	
Positive, n = 8	0	0.85 (0.82–0.89)	0.610
HPV 39			
Negative, n = 355	53 (14.9)	Reference	
Positive, n = 21	2 (9.5)	0.60 (0.14–2.65)	0.752
HPV 45			
Negative, n = 372	55 (14.8)	Reference	
Positive, n = 4	0	0.85 (0.82–0.89)	1.000
HPV 51			
Negative, n = 348	50 (14.4)	Reference	
Positive, n = 28	5 (17.9)	1.29 (0.47–3.57)	0.581
HPV 52			
Negative, n = 325	46 (14.2)	Reference	
Positive, n = 51	9 (17.6)	1.30 (0.59–2.85)	0.512
HPV 56			
Negative, n = 366	54 (14.8)	Reference	
Positive, n = 10	1 (10.0)	0.64 (0.80–5.17)	1.000
HPV 58			
Negative, n = 332	44 (13.3)	Reference	
Positive, n = 44	11 (25.0)	2.18 (1.03–4.63)	0.038
HPV 59			
Negative, n = 372	54 (14.5)	Reference	
Positive, n = 4	1 (25.0)	1.96 (0.20–19.22)	0.470
HPV 66			
Negative, n = 357	51 (14.3)	Reference	
Positive, n = 19	4 (21.1)	1.60 (0.51–5.01)	0.500
HPV 68			
Negative, n = 355	50 (14.1)	Reference	
Positive, n = 21	5 (23.8)	1.91 (0.67–5.44)	0.211
Biopsy (pieces)			
1, n = 182	17 (9.3)	Reference	
≥ 2, n = 194	38 (19.6)	2.36 (1.28–4.36)	0.005
Colposcopic impression, n = 374			
Low-grade lesion or normal, n = 325	33 (10.2)	Reference	
High-grade lesion, n = 49	22 (44.9)	6.98 (3.59–13.56)	< 0.001

*hr-HPV* High-risk human papillomavirus; *HSIL*^+^ High-grade squamous intraepithelial lesion or worse; *SCCA* Squamous cell carcinoma.

Among the 291 women who continued follow-up for at least six months, the initial pathology was one woman was diagnosed with invasive cancer, 40 women with HSIL, 120 women with LSIL, and 130 women with inflammation/normal cervical pathology. The median follow-up time was 41.8 months (IQR 26.5–54.0). Subsequent detection of SIL pathology occurred in 22 women (7.6%), including 14 cases of LSIL and 8 cases of HSIL (Table 3). The median time to subsequent detection of SIL was 28.7 months (IQR 14.99–41.7). When examining the median time to subsequent detection of HSIL, it was 31.5 months (IQR 17.8–53.6), and the median time to subsequent detection of LSIL was 19.0 months (IQR 13.7–28.3). However, no significant risk factors were identified in association with the subsequent detection of SIL. Additionally, the Cox proportional hazards model, which aimed to predict the time to recurrence, did not yield statistically significant results. Based on immediate pathology, the woman with invasive cancer was successfully treated without recurrence. Among the 40 women with immediate HSIL pathology, three showed subsequent detection of LSIL pathology during follow-up (ranging from 14.0 to 53.3 months), and one exhibited vaginal HSIL post-hysterectomy at 15.1 months of follow-up. Furthermore, among the 120 women initially diagnosed with LSIL, six women were subsequently detected LSIL within a follow-up period of 7.4 to 24.9 months, and four women were detected with HSIL within a follow-up period of 16.8 to 26.9 months. Among 130 women initially diagnosed with inflammation/normal cervical pathology, five showed subsequent LSIL pathology within a follow-up time range of 12.1–49.0 months and 3 HSIL pathology were detected in the follow-up period of 13.4 to 21.5 months (Table 3). It is important to note that all women in the study remained free of disease at the last visit date.

**Table 3:** Associated factors for 22 study women who had a subsequent occurrence of the squamous intraepithelial lesions out of 291 women who continued follow-up for ≥ 6 months.

Variables	Subsequent SIL N (%)	Pathology	
		14 LSIL	8 HSIL
Age (years)			
< 40, n = 127	12 (9.4)	9	3
≥ 40, n = 164	10 (6.1)	5	5
HPV status			
Negative hr-HPV, n = 102	6 (5.9)	5	1
Positive hr-HPV, n = 190	16 (8.4)	9	7
High-risk HPV genotyping			
HPV 16			
Negative, n = 239	17 (7.1)	12	5
Positive, n = 52	5 (9.6)	2	3
HPV 18			
Negative, n = 274	20 (7.3)	12	8
Positive, n = 17	2 (11.8)	2	0
HPV 31			
Negative, n = 280	21 (7.5)	14	7
Positive, n = 11	1 (9.1)	0	1
HPV 33			
Negative, n = 285	21 (7.4)	14	7
Positive, n = 6	1 (16.7)	0	1
HPV 35			
Negative, n = 285	22 (7.7)	14	8
Positive, n = 6	0	0	0
HPV 39			
Negative, n = 275	20 (7.3)	12	8
Positive, n = 16	2 (12.5)	2	0
HPV 45			
Negative, n = 287	22 (7.7)	14	8
Positive, n = 4	0	0	0
HPV 51			
Negative, n = 268	19 (7.1)	11	8
Positive, n = 23	3 (13.0)	3	0
HPV 52			
Negative, n = 252	18 (7.1)	12	6
Positive, n = 39	4 (10.3)	2	2
HPV 56			
Negative, n = 282	21 (7.4)	13	8
Positive, n = 9	1 (11.1)	1	0
HPV 58			
Negative, n = 260	19 (7.3)	14	5
Positive, n = 31	3 (9.7)	0	3
HPV 59			
Negative, n = 288	22 (7.6)	14	8
Positive, n = 3	0	0	0
HPV 66			
Negative, n = 278	21 (7.6)	14	7
Positive, n = 13	1 (7.7)	0	1
HPV 68			
Negative, n = 275	19 (6.9)	13	6
Positive, n = 16	3 (18.8)	1	2
Biopsy (pieces)			
1, n = 132	13 (9.8)	7	6
≥ 2, n = 159	9 (5.7)	7	2
Colposcopic impression			
Low-grade or normal, n = 252	18 (7.1)	11	7
High-grade, n = 39	4 (10.3)	3	1
Immediate pathology			
No SIL, n = 130	8 (6.2)	5	3
LSIL, n = 120	10 (8.3)	6	4
HSIL, n = 40	4 (10.0)	3	1
Invasive carcinoma, n = 1	0	0	0

*hr-HPV* High-risk human papillomavirus; *HSIL* High-grade squamous intraepithelial lesion; *IQR* Interquartile range; *LSIL* Low-grade squamous intraepithelial lesion; *SIL* Squamous intraepithelial lesion.

## Discussion

Cervical cancer is preventable, and cytology-based screening test has played a crucial role for decades. The most common cytology abnormality is ASC-US, accounting for 1.5–5.7% of all screening results^5,12,13,17^. Pathology diagnoses in women with ASC-US cytology include normal, cervicitis, LSIL, HSIL, or invasive cancer. The current study revealed that the proportion of immediate HSIL^+^ pathology in women with ASC-US was quite high, which aligns with previous studies reporting ranges of 5.1–17.0%^7,14,17–19^. A triage test for women with ASC-US before referral to colposcopy is necessary to counterbalance benefits and harm.

The randomized Atypical Squamous Cells of Undetermined Significance/Low-Grade Squamous Intraepithelial Lesion Triage Study (ALTS), which utilized hybrid capture® (HC) II in women with ASC-US, reported proportion of positive hr-HPV, immediate CIN 2^+^, and CIN 3^+^ of 50.6%, 11.4%, and 5.1%, respectively. The performance of hr-HPV in detecting CIN 2^+^ had a high sensitivity of 95.9% and a negative predictive value (NPV) of 98.9%^18^. Furthermore, hr-HPV testing is an economic strategy for classifying ASC-US with a colposcopy referral rate of 50.6%^20^. In addition to the ALTS study, the Kaiser Permanente Northern California (KPNC) research program conducted a similar investigation. In this cohort, the proportion of positive hr-HPV by HCII® in women with ASC-US was 48.4%, and immediate CIN 2^+^ was 7.7%. Among women with ASC-US, those positive for hr-HPV had a higher proportion of immediate CIN 2^+^ (14.8%) than those negative for hr-HPV (0.1%). Similarly, the proportion of immediate CIN 3^+^ in hr-HPV positive women was 4.9%, whereas it was 0.03% in hr-HPV negative women^19^.

Thus, from 2012 through to the most recent update of the 2019 ASCCP recommendations, two preferred management strategies for women with ASC-US cytology have emerged. The first strategy involves reflex hr-HPV testing, where a positive result leads to colposcopy, while a negative result permits HPV-based test in the next three years^6^. The alternative strategy suggests repeating cytology at one year. The WHO 2021 guidelines specifically recommend immediate colposcopy for women with ASC-US who test positive for hr-HPV^3^. None of the international guidelines have yet addresses the role of specific hr-HPV genotypes in triaging women with ASC-US cytology.

The higher the prevalence of hr-HPV infection, the higher the proportion of HSIL^+^ detection. Variations exist in the proportion of hr-HPV detection, which ranges from 32.0% to 85.3%, the proportion of initial HSIL^+^ pathology, and the diversity of hr-HPV genotyping across various studies of woman with ASC-US^12,13,15,17–19,21–24^. The ATHENA study, conducted with 1578 women with ASC-US using the cobas® test, found the proportion of positive hr-HPV to be 32.0% and the immediate CIN 2^+^ was 5.1% (or expressed as immediate CIN 3^+^, 2.9%). No cases of AIS or invasive cancer were diagnosed^16^. Three other studies presented the proportion of positive hr-HPV in women with ASC-US as 33.7%, 41.0%, and 67.2%. The immediate CIN 2^+^/HSIL^+^ pathology was reported at 7.3%, 7.4%, and 23.1%^13,14,25^. The NPV of the hr-HPV test was reported to be 100%^25^. The current study showed a proportion of positive hr-HPV consistent with the range of previous studies, and the hr-HPV test demonstrated the capability to predict immediate HSIL^+^ with a sensitivity of 90.6%, an NPV of 96.3%, and a colposcopy referral rate of 64.4%.

Each hr-HPV type delivers different risks for the development of CIN 2^+^ and varies geographically. The proportion of CIN 2^+^ in women with ASC-US who tested positive for hr-HPV was 13.3–35.6%^12,14,15,17,23^. ALTS reported that CIN 2^+^ in women with ASC-US who tested positive for hr-HPV was 26.2%, with the highest for HPV 16 positive women being 48.5%^23^. The ATHENA trial reported CIN 2^+^ in hr-HPV-positive women as 14.0%. They presented the risk of CIN 2^+^ in women positive for HPV 16, HPV 18, non-HPV 16/18, and negative for hr-HPV as 31.5%, 4.3%, 8.6%, and 0.8%, respectively^17^. A study of 1620 women with ASC-US found 75.3% to be positive for hr-HPV. And total immediate CIN 2^+^ prevalence was 33.6%, which broke down into positive hr-HPV 16/18 at 46.9% and hr-HPV non-16/18 at 30.3%^21^. Another study displayed a prevalence of CIN 2^+^ in ASC-US women who detected HPV 16, 18/45, another hr-HPV and negative hr-HPV at 31.6%, 10.9%, 6.4%, and 0.6%, respectively^24^. A retrospective study performed in China demonstrated that the common hr-HPV genotypes detected in ASC-US women were HPV 16, 52, 58, 18, 53/56, and 51, respectively. The proportion of HSIL^+^ was highest in women positive for HPV 16 (63.9%), followed by HPV 33, 51, 58, 52, and 18 (57.5%, 36.1%, 36.1%, 58.3%, and 26.4%), respectively^15^. Wang et al. reported HPV 52, 58, 16, 51, and 39 as the five most common hr-HPV genotypes detected in ASC-US women. They found the independent factor in predicting immediate CIN 2^+^ was HPV 16 (OR 37.38, 95% CI 20.58–67.88, *P* < 0.001). Other independent factors were HPV 58 (OR 6.97, 95% CI 3.35–14.48, *P* < 0.001), HPV 18 (OR 4.62, 95% CI 1.63–13.11, *P* = 0.004), HPV 52 (OR 4.49, 95% CI 2.42–8.31, *P* < 0.001), HPV 31 (OR 3.39, 95% CI 1.07–10.70, *P* = 0.038), and HPV 33 (OR 2.22, 95% CI 1.46–8.11, *P* = 0.043). The study also suggested using the HPV 16/18/31/33/52/58 model for triage in women with ASC-US to detect CIN 2^+^ with sensitivity, specificity, PPV, and NPV at 93.1%, 73.0%, 18.0%, and 99.4%, respectively, and a referral rate to colposcopy of 30.9%^12^. The current study found that the most common hr-HPV types in women with ASC-US were 16, 58, and 52, respectively. However, the highest proportion of HSIL^+^ was found in those infected with HPV types 16, 18, 58, and 59, respectively. These results, partially align with the ASCCP 2019 recommendations and suggest that hr-HPV genotyping should be integrated into triage management for women with ASC-US cytology.

In terms of the cumulative risk of CIN2^+^/CIN 3^+^ in women with ASC-US who tested positive for hr-HPV, a study by Schiffman et al. suggested that the 3-year cumulative risk of CIN 3^+^ was 5.2%. Additionally, the risk was 16.0%, 7.4%, 7.0%, and 7.1% in women who tested positive for HPV 16, HPV 18, HPV 31, or HPV 33/58, respectively. Therefore, women who carrying these five genotypes should be referred for immediate colposcopy, whereas those carrying other hr-HPV types could wait a year before retesting^26^. The ALTS reported the 2-year cumulative rates of CIN 2 and CIN 3 in women with ASC-US as 6.7% and 8.8%, respectively^27^. Furthermore, when stratified by hr-HPV testing, the 2-year cumulative risk of CIN 3^+^ in women with ASC-US carrying hr-HPV was 15%, and this rate increased to 32.5% if they were carrying HPV 16^23^. The KPNC cohort reported that the 5-year risk for developing a CIN3^+^ pathology in women with ASC-US who tested positive for hr-HPV was 6.8%, compared to 0.4% in those who tested negative^19,28^. Thus, the surveillance interval for women with ASC-US should be specified based on the genotyping of hr-HPV prevalent in of the individual country. Based on the genotyping of hr-HPV and the timing of subsequent SIL detection reported in the current study, surveillance by cytology or hr-HPV testing at 12, 24, 36, and 48 months could be suitable.

Age has been previously reported as a significantly associated factor with an increased risk of developing CIN 2^+^ pathology. A study in New York of 2145 women with ASC-US who tested positive for the Aptima HPV mRNA test and underwent colposcopic directed biopsy found that the proportion of CIN 2^+^ was 8.8%. When considering age groups, the highest incidence of CIN 2^+^ was found in those younger than 25 years compared to the age group 50–59 years, with rated of 15.4% versus 4.8%, respectively^29^. The highest detection proportion of HSIL^+^ was in those aged ≤ 30 years (40.5%) and the lowest in the age group 51–60 years (21.7%). Invasive cancer was most often detected in those aged > 60 years (3.4%) and second most in the age group 51–60 years (2.7%). This is similar to the current study, which found HSIL^+^ to be more prevalent in individuals < 40 years and invasive cancer was detected more often in those older than 40 years^15^. On the contrary, the authors reported a positive trend with age groups of < 30 years, 30–44 years, and ≥ 45 years, in relation to the risk of non-HPV 16/18 CIN 3 (23.6% vs 32.1% vs 38%, *P* < 0.001)^30^.

Optimal integrated screening and classification strategy for selected women at highest risk of having HSIL^+^ pathology to refer for colposcopy at the right time is necessary. Strategies for managing ASC-US with other secondary tests, such as E6/7 mRNA or p16/Ki67 dual stain, have been proposed to identify these women. The European Equivocal or Mildly Abnormal Pap Cytology Study (EEMAPS) reported that the proportion of positive p16/Ki-67 in ASC-US women who detected CIN 2^+^ was 20% compared to positive hr-HPV which was 69.5%. Moreover, p16/Ki-67 had a higher performance for CIN 2^+^ detection with a sensitivity and specificity of 92.2% (hr-HPV 90.9%) and 80.6% (hr-HPV 36.3%), respectively^31^. The Primary ASC-US LSIL Marker Study (PALMS) stated that p16/Ki-67 had a comparable sensitivity but higher specificity compared to HCII® for CIN 2^+^ detection in the ASC-US subgroup^32^. Zhu et al., compared the performance of three tests; the p16/Ki67, the hr-HPV DNA testing and the HPV E6/E7 RNA test, for detection CIN 2^+^ in women with ASC-US. They stated that the sensitivity was 98.2%, 98.2%, and 87.0%, respectively. Furthermore, the specificity was 82.5%, 17.5%, and 42.7%, respectively^22^. Further studies should be conducted to determine factors, such as, p16/Ki67 or DNA methylation, that could be added to the selection criteria to identify women with ASC-US for the highest benefit and the best cost-effective protocols for colposcopy.

The strength of the current study is the prospective study that collected complete clinical data, colposcopy findings, hr-HPV testing, and pathology diagnosis were achieved within one month of the cytology examination date and have long-term follow-up data. The drawback of the current study is the fact that we did not measure the quantitative assay of hr-HPV or HPV E6/E7 mRNA testing, we did not evaluate inter- or intra-observer variation among the three colposcopists, and we did not perform cost-utility analysis.

In conclusion, hr-HPV genotyping may play a role in the immediate management and follow-up schedule for women with ASC-US.

## Data Availability

Correspondence and requests for materials should be addressed to I.R.
